# The Effect of Periprocedural Clinical Factors Related to the Course of STEMI in Men and Women Based on the National Registry of Invasive Cardiology Procedures (ORPKI) between 2014 and 2019

**DOI:** 10.3390/jcm10235716

**Published:** 2021-12-06

**Authors:** Janusz Sielski, Karol Kaziród-Wolski, Karolina Jurys, Paweł Wałek, Zbigniew Siudak

**Affiliations:** 1 Collegium Medicum, Jan Kochanowski University in Kielce, Żeromskiego 5 St., 25-600 Kielce, Poland; JSIELSKI7@interia.pl (J.S.); kk-wolski@wp.eu (K.K.-W.); zbigniew.siudak@gmail.com (Z.S.); 2 Hospital Emergency Department, Provincial Hospital, 25-736 Kielce, Poland; karolina.jurys@gmail.com

**Keywords:** myocardial infarction, acute coronary syndrome, percutaneous coronary intervention

## Abstract

Background: There are several sex-related differences in the course, management, and outcomes of ST-elevation myocardial infarction (STEMI). This study aimed to identify the risk factors that may affect the odds of procedure-related death in patients with STEMI. Methods: The observational cohort study group consisted of 118,601 participants recruited from the National Registry of Invasive Cardiology Procedures (ORPKI). Results: Procedure-related death occurred in 802 (1.0%) men and in 663 (1.7%) women. The odds of procedure-related death among women were significantly higher than among men (OR, 1.76; 95% CI, 1.59–1.95; *p* < 0.001). The probability of procedure-related mortality was highest in both men and women with cardiac arrest in the cath lab, critical stenosis of the left main coronary artery, and direct transfer to the cath lab. The factors that reduced the probability of procedure-related mortality in both men and women were thrombolysis in myocardial infarction (TIMI) flow grade and the use of P2Y12 inhibitors in the peri-infarct period. Psoriasis was associated with increased odds of procedure-related death among men, whereas cigarette smoking reduced the odds among women. Conclusions: Procedure-related deaths occurred more frequently in women than men with STEMI. Additional scrutiny needs to be undertaken to identify factors influencing survival regarding gender differences.

## 1. Introduction

Traditional population-based studies have shown that women have higher in-hospital mortality rates than men with ST-elevation myocardial infarction (STEMI). This trend is most visible in young women, whereas in older age groups the gender gap in mortality narrows [1,2]. More recent studies have provided more evidence confirming sex-based differences, not only in 30-day mortality rates after STEMI but also in the development of heart failure after myocardial infarction [3,4]. Furthermore, the evidence indicates gender differences in STEMI management: the use of dual antiplatelet therapy and heparin during hospitalization and of angiotensin-converting-enzyme (ACE) inhibitors, beta-blockers, and statins at discharge [5,6].

As in any invasive procedure, there is always some risk associated with cardiac catheterization, so it is essential to both the patient and the physician to have appropriate indications for a procedure or medication. Periprocedural myocardial infarction or the need for emergency cardiac surgery are important complications of percutaneous coronary intervention [7,8].

Complications may always arise as a result of treatment. These complications may be fatal. Mortality is frequently defined as 30-day, in-hospital, or long-term mortality, whereas perioperative mortality is the term used in patients undergoing surgical interventions. Procedure-related death is most often associated with general anesthesia and adverse reactions to anesthetics [9]. In 2019, in Poland a total of 172,521 percutaneous coronary interventions were performed, 20% of which were in STEMI patients [10]. Such interventions may result in death. Procedure-related death is also a good marker of the impact of various factors on the course of the procedure, on both the side of the patient (risk factors) and the physician [11]. Studies of numerous groups of patients show significant differences in the clinical outcomes of the acute coronary syndrome, especially STEMI. Women experience longer delays in administering the procedure and higher 30-day mortality compared to men. In contrast, women with NSTEMI did not have these delays and there were no differences in the mortality rates compared to men [12]. In addition, studies also found differences in symptoms between women and men. Women misinterpreted significant disease symptoms much more often [13]. Additionally, as shown by Shah et al. in their meta-analysis of 56 studies on in-hospital management in STEMI (705,098 patients, including 31% women), in-hospital mortality was higher in women than in men. Additionally, higher frequencies of repeated myocardial infarction, stroke, and major bleeding were found in the group of women [14]. In turn, Eindhoven et al. in a large study involving 59,534 patients, found significant differences in the prognosis for women compared to men, resulting from the tendency for non-compliance with medical recommendations among the women [15].

In this study, procedure-related death was used as one of the indicators of the impact of these various factors on the course of the procedure based on a large national registry (the National Registry of Invasive Cardiology Procedures [ORPKI]) [16,17].

The purpose of the study is to identify the most important factors that may affect the odds of procedure-related death in patients with STEMI.

## 2. Materials and Methods

**Figure 1:**
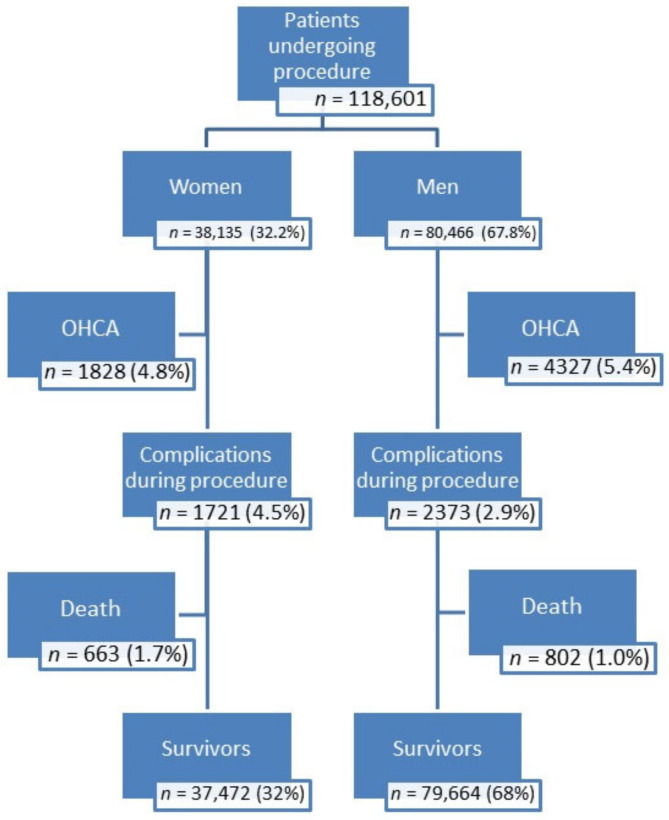
General flow chart of patients. OHCA—out of hospital cardiac arrest.

Large national registries that collect data over long periods of time provide important insight into disease states. Accumulating large amounts of data on various diseases and specific treatments guarantees the correctness of the statistical analysis. ORPKI is one such large database. The registry was launched in 2004 by the Association of Cardiovascular Interventions of the Polish Cardiac Society. Now the registry is coordinated by the Jagiellonian University Medical College in Krakow, collecting data from 161 cath labs around the country. For the purposes of this study, data from 2014 to 2019 were extracted from the database. The study population (134,346 patients) consisted of patients admitted to the hospital in compliance with the guidelines of the Polish Cardiac Society and selected for the invasive treatment of acute coronary syndromes with persistent elevation of the ST segment. The variables with missing values were as follows: time from first contact to inflation (15,483 patients), time from pain to inflation (16,316 patients), time from pain to first contact (16,078 patients), and total radiation dose during the procedure (5208 patients). Finally, after excluding cases with missing data the study group consisted of 118,601 participants. The study population was divided into two groups: males vs. females. They were evaluated with respect to comorbidities, predisposing factors, and the medication used in STEMI. The following risk factors for STEMI were identified in the study groups: conventional risk factors, prehospital factors, antiplatelet drugs/anticoagulants and procedure-related risk factors, coronary anatomy, and complications. Figure 1 includes a general flow chart of the studied population.

The prevalence of different characteristics of the study population was estimated, and two separate regression analyses were performed, one for men and one for women. Multiple logistic regression analysis was used to evaluate the factors that might affect the occurrence of procedure-related death in the cath lab. The study was carried out in accordance with the ethical standards of the 1964 Declaration of Helsinki and informed consent was obtained from each patient for coronary angiography before the invasive procedure. Due to the nature of the registry and the anonymity of the data, consent to be listed in the registry was not obtained. Additionally, because the study used anonymous data, it was exempt from the bioethics committee approval.

### 2.1. Patient and Public Involvement

The patients and the public were not involved in the design, conduct, reporting, or dissemination plans of this research.

### 2.2. Statistical Analysis

The quantitative variables are expressed as median (inter-quartile range). Categorical variables are presented as numbers and percentages. The normality of data distribution was tested with the Kolmogorov–Smirnov test. The χ2 test was used to test the interdependence of pairs of variables for double classifications. The Mann–Whitney test was used for non-normally distributed variables to assess within-group differences. The logistic regression models were used to estimate odds ratios with 95% confidence intervals and *p* values. Missing values in the quantitative column were replaced with a median because of the non-normal distribution. A *p*-value less than 0.05 was considered significant. Statistical analysis was performed using Med-Calc Statistical Software, version 19.7 [18].

**Figure 2:**
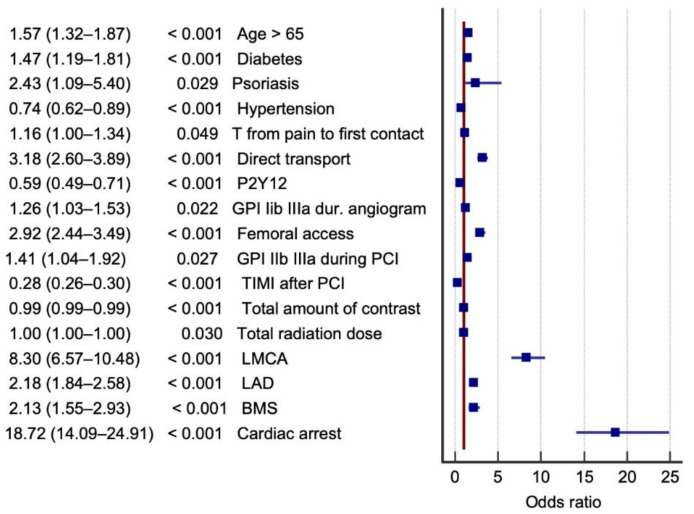
Factors that affect the probability of procedure-related mortality among men. Legend: BMS, bare metal stent; MI, myocardial infarction; GPI IIb/IIIa, IIb/IIIa, glycoprotein inhibitor; LAD, critical stenosis of left anterior descending artery; LMCA, critical stenosis of left main coronary artery; PCI, percutaneous coronary intervention; TIMI, thrombolysis in myocardial infarction.

**Figure 3:**
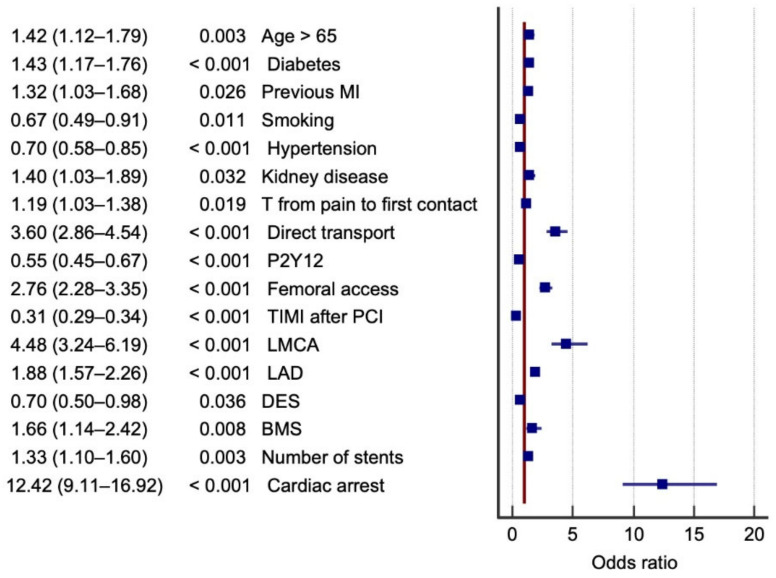
Factors that affect the probability of procedure-related mortality among women. Legend: BMS, bare metal stent; DES, drug-eluting stent; MI, myocardial infarction; LAD, critical stenosis of left anterior descending artery; LMCA, critical stenosis of left main coronary artery; PCI, percutaneous coronary intervention; TIMI, thrombolysis in myocardial infarction.

The logistic regression model was based on the following scheme. Procedure-related death was considered a dependent variable. The variables were divided into three groups according to shared characteristics: factors related to clinical characteristics and prehospital management (Table 1); pharmacological and periprocedural factors (Table 2); and coronary anatomy, implanted stents, and complications during the procedure (Table 3). Cases of patients with STEMI evolution—without indications for urgent coronary angiography—were added to the ORPKI register. Since there are records that have been in the registry for a very long time, the time variables were divided into 3 categories (<12 h, 12–48 h, and >48 h), in accordance with the European Society of Cardiology (ESC) guidelines. Multiple logistic regression was used to evaluate these groups of factors for both sexes (Table 4, Table 5 and Table 6). Each table (Table 4, Table 5 and Table 6) was a separate model of multivariate logistic regression. The factors in each table were adjusted to each other. Then, two separate regression analyses of the statistically significant results for each group of factors were performed, one in the men and one in the women; the results are presented on tree diagrams (Figure 2 and Figure 3, Tables S1 and S2, Supplementry Materials). Tables S3–S5 (Supplementry) show the descriptive characteristics of dead patients.

## 3. Results

The study population consisted of 118,601 patients admitted to the hospital with STEMI to undergo invasive cardiac procedures. There were 80,466 men and 38,135 women. The STEMI-related factors were divided into three groups: Group 1 encompassed conventional risk factors for STEMI and pre-hospital factors, such as conventional time intervals from the onset of chest pain, direct transfer to the cath lab, and out-of-hospital cardiac arrest (Table 1); Group 2 had factors such as the use of antiplatelet drugs/anticoagulants, procedure-related factors such as vascular access, TIMI flow grade, contrast dose, radiation exposure, etc. (Table 2); and Group 3 involved coronary anatomy, characteristics of the implanted stents, and catheterization-related complications (Table 3).

Procedure-related death occurred in 802 (1.0%) men and in 663 (1.7%) women. These results led us to look at the underlying causes of the disparity in procedure-related mortality among men and women. The odds of procedure-related death among women are significantly higher (OR, 1.76; 95% CI, 1.59–1.95; *p* < 0.001) than among men. Table 4, Table 5 and Table 6 summarize the results of multiple logistic regression analysis separately among men and women. The factors in each table were adjusted to each other.

All statistically significant variables in each group were entered into the regression model (Tables S1 and S2, Supplementary Materials). The probability of procedure-related mortality was highest in both men and women with cardiac arrest in the cath lab, critical stenosis of the LMCA, and direct transfer to the cath lab. The factors that reduced the probability of procedure-related mortality in both men and women were TIMI flow grade, the use of P2Y12 inhibitors in the peri-infarct period, and arterial hypertension (Figure 2 and Figure 3). However, there was a significant difference in the probability of cardiac arrest in the cath lab between STEMI men and women. Differences in procedure-related mortality were related mainly to a history of psoriasis, which significantly increased the likelihood of death in the cath lab only among men (among women, the parameter did not attain statistical significance and the data are not included in Figure 3), and smoking, which reduced procedure-related mortality only in women (Figure 3). The impact of selected variables on procedure-related mortality is displayed in detail in Figure 2 and Figure 3, where only significant results are included. The ORPKI registry does not specify where the P2Y12 inhibitor was administered. Most patients in Poland go to the cath lab directly from an ambulance. Thus, the first administration of P2Y12 drugs is in the ambulance, prior to admission to the hospital for STEMI. Clopidogrel was administered in most cases.

## 4. Discussion

Registry studies aggregating large datasets are very valuable scientifically, almost as much as randomized studies and meta-analyses. The ORPKI registry is a classic example of a multicenter database. Identifying similarities and differences between men and women with STEMI is a very important aspect of research in this field for detecting associated risk and choosing an appropriate treatment strategy.

There are a number of observational studies which compare in-hospital mortality in men and women with STEMI. In a large observational study involving 23 809 patients with STEMI between 2013 and 2015, Hannan et al. compared in-hospital mortality for men and women. The investigators found that women had higher in-hospital mortality rates, especially among patients aged 65 and older [19].

A population-based registry, the Swedish Web System for Enhancement and Development of Evidence-Based Care in Heart Disease Evaluated According to Recommended Therapies (SWEDEHEART), involving 216,512 patients hospitalized between 2003 and 2013, showed higher mortality among women than men in one study arm [20]. A Spanish retrospective longitudinal study of 445,145 episodes of myocardial infarction from 2005 to 2015 showed that women had an increased risk of in-hospital mortality, with higher mortality for STEMI and lower for NSTEMI [21]. In the present study, using another large national registry of 118,601 patients with STEMI, women had higher procedure-related mortality than men. In a large study, the Coronary Angiography and PCI Registry of the German Society of Cardiology, conducted between 2007 and 2009 and involving 185,312 patients undergoing coronary artery surgery due to acute coronary syndrome (including 27.9% women), Heer et al. found hospital mortality after coronary artery surgery in STEMI to be 20% higher in women than in men. However, there was no difference in mortality between the groups after NSTEMI coronary artery surgery due to cardiogenic shock [22]. Similarly, Lee et al. presented an analysis from 24 hospitals with a total of 35,322 patients (including 20.56% women) covering the period 2012 to 2016. The women in the study group were older and more often had diabetes, hypertension, heart failure, and renal failure than the men. Among the women, there was a higher rate of in-hospital, 30-day, and annual mortality in both the STEMI and NSTEMI groups [23]. Women have a higher risk for thrombotic and bleeding events compared to men. The higher mortality in women can be explained by gender-specific differences in platelet function, hormones, vascular factors, and coagulation mechanisms, which directly affect antiplatelet therapy. However, there is still no strong evidence explaining this dependency [24,25].

It has been known for years that patients with critical stenosis in the left main coronary artery (LMCA) are at increased risk of in-hospital mortality. Ali et al. studied 312 patients with STEMI and found significantly higher in-hospital mortality among those with culprit lesions in the LMCA [26]. Critical LMCA stenosis can especially be found in elderly patients who, because of their advanced age, are frequently not added to large databases. In a single-center retrospective study, Sliman et al. analyzed 139 patients aged 80 and older who underwent either PCI or CABG. Most patients in the study population had multi-vessel coronary artery disease and critical lesions in the LMCA [27].

Patient transfer time to a cath lab is another factor that may significantly affect in-hospital mortality. To reduce mortality, current ESC and PCS guidelines on STEMI recommend developing systems to minimize transfer-related time delays in initiating STEMI treatment [28,29,30]. Our large study of STEMI patients receiving treatment at various Polish centers between 2014 and 2019 demonstrated higher mortality in the cath lab both among men and women who were transferred directly for coronary intervention. This may be because such patients were in a poorer state, which had to be considered when deciding on direct transfer.

Reduced periprocedural mortality rates in STEMI were reported after coronary angioplasty or during treatment with P2Y12 inhibitors. These results are similar for men and women. In a large analysis of patients with STEMI and multi-vessel disease undergoing coronary intervention (the complete revascularization group; *n* = 2016), Metha et al. demonstrated that the use of P2Y12 inhibitors was not associated with in-hospital mortality [31]. In contrast, older patients (aged 75 and older; *n* = 1862) randomized to the ATLANTIC trial who were receiving P2Y12 inhibitors in pre-hospital management of STEMI were characterized by higher mortality and less successful mechanical reperfusion [32].

TIMI flow grade after coronary reperfusion is an interesting factor that may be related to periprocedural mortality in STEMI. In a large study of 28,421 STEMI patients recruited between 2005 and 2010 from a nationwide registry in Korea, Park et al. demonstrated that TIMI flow grade was one of the independent predictors of cardiac death [33]. In our study, TIMI flow grade and early use of P2Y12 inhibitors were found to reduce the risk of death in the cath lab in both men and women with STEMI. In the Polish STEMI population, clopidogrel, ticagrelor, and prasugrel were the most prevalent over the study period (69.0%, 10.1%, and 1.1%, respectively) [34].

Cigarette smoking is a well-known risk factor for STEMI; however, the smokers’ paradox was described in the literature. Redfords et al. analyzed 10 randomized studies with STEMI patients undergoing primary PCI (*n* = 2564). The smokers had lower in-hospital mortality and shorter hospital stay. The investigators ascribed this paradox to the younger age of the patients and their fewer comorbidities [35]. Other investigators have confirmed the smokers’ paradox [36]. The present study, carried out in a large group of patients with STEMI (*n* = 118,601), corroborates the occurrence of the smokers’ paradox, but only among women. Cigarette smoking has long been recognized as a risk factor for cardiovascular disease, including acute coronary syndrome [37]. However, some studies have shown that smoking may play a beneficial role in STEMI and may prolong short-term survival [38,39]. This phenomenon is called “the smoker’s paradox” and is influenced by many factors, including lower platelet activity in smokers regardless of the type of P2Y12 inhibitor used in early treatment [40]. The literature also includes studies that show a similar prognosis in chronic smokers and non-smokers. An analysis by De Luca et al. included 830 patients with STEMI undergoing coronary artery surgery. The infarct area was determined by scintigraphy 30 days after the procedure. In smokers, MI was associated with younger age, male sex, hypertension, diabetes, and a shorter time from the onset of coronary pain. In the results, it was emphasized that the smokers’ infarction was not associated with a larger infarct area as described by scintigraphy [41]. The morphological basis of this phenomenon is puzzling; there are various assumptions on the subject.

The differences in the local reaction in smokers are attributed to their greater tendency towards clot formation in myocardial ischemia and their significantly better response to antiplatelet therapy [42,43,44]. Thus, the phenomenon of the smoker’s paradox is still not fully understood; we hope that the results of our research will be an important contribution to the discussion of this problem.

Frailty in old age is a known factor in deteriorating the periprocedural prognosis in STEMI and increasing in-hospital mortality. Calvo E et al., in a prospective study of 259 fragile STEMI patients, found that frailty was associated with diabetes, hypertension, a history of ischemic stroke, and higher in-hospital mortality [45]. Large databases of retrospective studies on large populations also confirm this relationship. In the large Australian CONCORDANCE registry (Australian Cooperative National Registry of Acute Coronary Care, Guideline Adherence and Clinical Events), Ashish et al. confirmed the correlation of frailty as a factor that deteriorates the prognosis in patients with STEMI. This study included 3944 MI patients aged 65 years and older who were treated at 41 Australian hospitals from 2009 to 2016. Patients with frailty confirmed in earlier studies had higher general-cause mortality after discharge from the hospital, although cardiac mortality was the same as in the control population [46]. Similarly, the study by Yoshioka K. et al. on 354 patients with STEMI (mean age: 69.8 years) who underwent percutaneous intervention between 2014 and 2017 confirmed that significant frailty is associated with mid-term prognosis in STEMI [47].

Psoriasis is another factor that was found to have a significant impact on prognosis in patients with STEMI. Psoriatic disease affects the skin, but blood vessels can also become inflamed. Siudak et al. made very important observations about psoriasis in a study of 405,078 patients recruited from the ORPKI database. This skin condition was diagnosed in 1507 (0.4%) participants. Psoriasis was an independent predictor of allergic reactions, but there was no proof of a higher risk of death in the cath lab. Moreover, in the presence of psoriasis as a comorbid condition, patients are generally characterized by chronic inflammation and have a higher cardiovascular risk than in the general population. It is correlated with the deterioration of COVID-19 prognosis after acute coronary syndrome [48]. In our study (*n* = 118,601), there was such a relationship among men 

Large, registry-based studies in STEMI, by collecting large amounts of data, can identify variables that are common among men and women as well as those that differentiate the sexes. These changes can be found in retrospective (large registries) and prospective studies. In time, new elements will appear, for instance, those related to environmental pollution or the COVID-19 pandemic.

## 5. Conclusions

1. In the present study, procedure-related death occurred more frequently in women with STEMI than men with STEMI.
2. Differences in perioperative mortality of women and men were found. The most important elements are smoking and psoriasis.
3. Although there are gender differences in peri-procedural conduct in patients with STEMI, the treatment of women and men in the cath lab should be the same and in accordance with the procedures

## Figures and Tables

**Table 1:** Factors related to clinical characteristics and prehospital management.

Variable	Total *n* = 118,601	Women *n* = 38,135 (32.2%)	Men *n* = 80,466 (67.8%)	*p* Value
Clinical factors				
Sex	118,601	38,135 (32.2)	80,466 (67.8)	<0.001
Age, median (Q1–Q3)	65 (57–74)	70 (62–80)	63 (56–70)	<0.001
Age (>65 years)	55,477 (46.8)	24,141 (63.3)	31,336 (38.9)	<0.001
Diabetes (*n*, %)	21,183 (17.9)	8956 (23.5)	12,227 (15.2)	<0.001
Previous stroke (*n*, %)	3907 (3.3)	1550 (4.1)	2357 (2.9)	<0.001
Previous MI (*n*, %)	14,813 (12.5)	4021 (10.5)	10,792 (13.4)	<0.001
Previous PCI (*n*, %)	13,763 (11.6)	3593 (9.4)	10,170 (12.6)	<0.001
Previous CABG (*n*, %)	2103 (1.8)	469 (1.2)	1634 (2.0)	<0.001
Smoking (*n*, %)	35,774 (30.2)	8240 (21.6)	27,534 (34.2)	<0.001
Psoriasis (*n*, %)	554 (0.5)	145 (0.4)	409 (0.5)	0.003
Hypertension (*n*, %)	70,855 (59.7)	24,688 (64.7)	46,167 (57.4)	<0.001
Kidney disease (*n*, %)	4050 (3.4)	1809 (4.7)	2241 (2.8)	<0.001
COPD (*n*, %)	1981 (1.7)	668 (1.8)	1313 (1.6)	0.13
Prehospital management				
Time from pain to first contact (*n*, %)				<0.001
<12 h	98,711 (83.2)	31,026 (81.4)	67,685 (84.1)	
12–48 h	14,905 (12.6)	5378 (14.1)	9527 (11.8)	
≥48 h	4985 (4.2)	1731 (4.5)	3254 (4.0)	
Time from pain to inflation or angiogram (*n*, %)				<0.001
<12 h	114,363 (96.4)	36,648 (96.1)	77,715 (96.6)	
12–48 h	2963 (2.5)	1045 (2.7)	1918 (2.4)	
≥48 h	1275 (1.1)	442 (1.2)	833 (1.0)	
Time from first contact to inflation or angiogram (*n*, %)				<0.001
<12 h	105,164 (88.7)	33,367 (87.5)	71,797 (89.2)	
12–48 h	10,032 (8.5)	3597 (9.4)	6435 (8.0)	
≥48 h	3405 (2.9)	1171 (3.1)	2234 (2.8)	
Direct transfer to cath lab (*n*, %)	29,216 (24.6)	9026 (23.7)	20,190 (25.1)	<0.001
OHCA (*n*, %)	6155 (5.2)	1828 (4.8)	4327 (5.4)	<0.001

CABG, coronary aortic bypass grafting; COPD, chronic obstructive pulmonary disease; MI, myocardial infarction; OHCA, out of hospital cardiac arrest; PCI, percutaneous coronary intervention.

**Table 2:** Pharmacological and periprocedural factors.

Variable	Total *n* = 118,601	Women *n* = 38,135 (32.2%)	Men *n* = 80,466 (67.8%)	*p* Value
Pharmacological factors				
ASA (*n*, %)	95,312 (80.4)	30,497 (80.0)	64,815 (80.5)	0.019
UFH (*n*, %)	105,172 (88.7)	33,778 (88.6)	71,394 (88.7)	0.44
LMWH (*n*, %)	4374 (3.7)	1361 (3.6)	3013 (3.7)	0.13
P2Y12 (*n*, %)	99,447 (83.9)	31,790 (83.4)	67,657 (84.1)	0.002
Thrombolysis (*n*, %)	112 (0.09)	42 (0.1)	70 (0.09)	0.23
GPI IIb/IIIa during angiogram (*n*, %)	29,737 (25.1)	8317 (21.8)	21,420 (26.6)	<0.001
Bivalirudin (*n*, %)	60 (0.05)	23 (0.06)	37 (0.05)	0.31
Periprocedural factors				
IVUS (*n*, %)	249 (0.2)	63 (0.2)	186 (0.2)	0.021
OCT (*n*, %)	56 (0.05)	13 (0.03)	43 (0.05)	0.15
Vascular access (*n*, %)				<0.001
radial	86,393 (72.8)	25,765 (67.6)	60,628 (75.3)	
femoral	32,208 (27.2)	12,370 (32.4)	19,838 (24.7)	
FFR (*n*, %)	68 (0.06)	17 (0.04)	51 (0.06)	0.21
Aspiration thrombectomy (*n*, %)	15,744 (13.3)	4618 (12.1)	11,126 (18.8)	<0.001
Rotablation (*n*, %)	75 (0.06)	24 (0.06)	51 (0.06)	0.98
GPI IIb/IIIa during PCI (*n*, %)	7297 (6.2)	2090 (5.5)	5207 (6.5)	<0.001
TIMI before PCI (*n*, %)				0.003
0	69,383 (58.5)	22,052 (57.8)	47,331 (58.8)	
1	17,550 (14.8)	5814 (15.2)	11,736 (14.6)	
2	17,131 (14.4)	5512 (14.5)	11,619 (14.4)	
3	14,537 (12.3)	4757 (12.5)	9780 (12.2)	
TIMI after PCI (*n*, %)				<0.001
0	2796 (2.4)	1119 (2.9)	1677 (2.1)	
1	1810 (1.5)	728 (1.9)	1082 (1.3)	
2	5708 (4.8)	2040 (5.4)	3668 (4.6)	
3	107,968 (91.3)	34,135 (89.9)	73,833 (92.0)	
Total amount of contrast, mL, median (Q1–Q3)	150 (120–200)	150 (120–200)	160 (130–200)	<0.001
Total radiation dose, mGy, median (Q1–Q3)	766 (437–1300)	643 (359–1108)	831 (482–1392)	<0.001

ASA, acetylsalicylic acid; FFR, fractional flow reserve; GPI IIb/IIIa, IIb/IIIa, glycoprotein inhibitor; IVUS, intravascular ultrasonography; LMWH, low molecular weight heparin; PCI, percutaneous coronary intervention; TIMI, thrombolysis in myocardial infarction; UFH, unfractionated heparin.

**Table 3:** Coronary anatomy, implanted stents, and complications during the procedure.

Variable	Total *n* = 118,601	Women *n* = 38,135 (32.2%)	Men *n* = 80,466 (67.8%)	*p* Value
Coronary anatomy and implanted stents				
RCA (*n*, %)	47,444 (40.0)	15,740 (41.3)	31,704 (39.4)	<0.001
LMCA (*n*, %)	2638 (2.2)	797 (2.1)	1841 (2.3)	0.031
LAD (*n*, %)	48,589 (41.0)	15,683 (41.3)	32,906 (40.9)	0.45
SvG (*n*, %)	668 (0.6)	125 (0.3)	543 (0.7)	<0.001
LIMA/RIMA (*n*, %)	93 (0.08)	27 (0.07)	66 (0.08)	0.52
Bifurcation (*n*, %)	8299 (7.0)	2461 (6.50	5838 (7.3)	<0.001
DES (*n*, %)	102,460 (86.4)	32,299 (84.7)	70,161 (87.2)	<0.001
BVS (*n*, %)	587 (0.5)	137 (0.4)	450 (0.6)	<0.001
BMS (*n*, %)	7098 (6.0)	2517 (6.6)	4581 (5.7)	<0.001
Number of implanted stents (*n*, %)				<0.001
0	9054 (7.6)	3385 (8.9)	5669 (7.0)	
1	90,135 (76.0)	28,455 (74.6)	61,680 (76.7)	
2	16,434 (13.9)	5311 (13.9)	11,123 (13.8)	
3	2978 (2.5)	984 (2.6)	1994 (2.5)	
DEB (*n*, %)	706 (0.6)	234 (0.6)	472 (0.6)	0.57
Complications during the procedure				
Total (*n*, %)	4094 (3.5)	1721 (4.5)	2373 (2.9)	<0.001
Death (*n*, %)	1465 (1.2)	663 (1.7)	802 (1.0)	<0.001
Cardiac arrest (*n*, %)	927 (0.8)	366 (1.0)	561 (0.7)	<0.001
Stroke (*n*, %)	18 (0.02)	11 (0.03)	7 (0.009)	0.009
Dissection (*n*, %)	146 (0.1)	73 (0.2)	73 (0.09)	<0.001
Bleeding at the puncture site (*n*, %)	52 (0.04)	26 (0.07)	26 (0.03)	0.006
Allergic reaction (*n*, %)	12 (0.01)	8 (0.02)	4 (0.005)	0.01
No reflow (*n*, %)	1756 (1.5)	692 (1.8)	1064 (1.3)	<0.001
Coronary artery perforation (*n*, %)	233 (0.2)	117 (0.3)	116 (0.1)	<0.001

BMS, bare metal stent; BVS, bioresorbable vascular scaffold; DEB, drug-eluting balloon; DES, drug-eluting stent; LAD, critical stenosis of left anterior descending artery; LMCA, critical stenosis of left main coronary artery; LIMA/RIMA, critical stenosis of left internal mammary artery/right internal mammary artery; RCA, critical stenosis of right coronary artery; SvG, critical stenosis of saphenous vein graft.

**Table 4:** Clinical and prehospital management factors affecting periprocedural mortality.

Variable	Women			Men		
	OR	95% CI	*p* Value	OR	95% CI	*p* Value
Age (>65 years)	1.99	1.60–2.47	<0.001	1.95	1.66–2.28	<0.001
Diabetes	1.52	1.26–1.83	<0.001	1.66	1.38–1.99	<0.001
Previous stroke	1.29	0.93–1.80	0.13	1.68	1.25–2.28	<0.001
Previous MI	2.02	1.53–2.67	<0.001	1.68	1.31–2.16	<0.001
Previous PCI	0.73	0.53–1.01	0.06	0.85	0.65–1.12	0.24
Previous CABG	0.95	0.48–1.88	0.88	0.82	0.51–1.33	0.43
Smoking	0.55	0.41–0.73	<0.001	0.77	0.64–0.92	0.003
Psoriasis	2.01	0.73–5.54	0.18	2.36	1.14–4.89	0.02
Hypertension	0.60	0.51–0.72	<0.001	0.65	0.56–0.76	<0.001
Kidney disease	1.58	1.20–2.10	0.001	1.61	1.20–2.18	0.002
COPD	1.08	0.61–1.90	0.8	1.51	0.98–2.31	0.06
Time from pain to first contact	1.56	1.35–1.81	<0.001	1.62	1.41–1.86	<0.001
Time from pain to inflation or angiogram	0.87	0.66–1.16	0.34	0.89	0.64–1.25	0.52
Time from first contact to inflation or angiogram	1.0002	0.99–1.0006	0.22	0.99	0.99–1.002	0.35
Direct transfer to cath lab	1.37	1.14–1.64	<0.001	1.16	0.98–1.36	0.09
OHCA	6.6	5.47–8.15	<0.001	9.08	7.71–10.69	<0.001

CABG, coronary aortic bypass grafting; COPD, chronic obstructive pulmonary disease; MI, myocardial infarction; OHCA, out of hospital cardiac arrest; PCI, percutaneous coronary intervention.

**Table 5:** Pharmacological and periprocedural factors affecting periprocedural mortality.

Variable	Women			Men		
	OR	95% CI	*p* Value	OR	95% CI	*p* Value
ASA	0.96	0.75–1.22	0.74	1.06	0.86–1.32	0.58
UFH	1.24	0.95–1.63	0.11	1.09	0.87–1.39	0.43
LMWH	*			*		
P2Y12	0.47	0.38–0.59	<0.001	0.44	0.36–0.54	<0.001
Thrombolysis	4.09	0.92–18.14	0.06	3.04	0.71–13.07	0.14
GPI IIb/IIIa during angiogram	1.14	0.92–1.41	0.22	1.36	1.13–1.63	<0.001
Bivalirudin	*			3.33	0.37–30.33	0.29
IVUS	*			0.62	0.08–4.71	0.64
OCT	*			3.43	0.43–27.59	0.25
Femoral access	4.06	3.38–4.87	<0.001	4.80	4.09–5.63	<0.001
FFR	*			4.43	0.59–33.09	0.15
Aspiration thrombectomy	1.08	0.83–1.41	0.57	1.17	0.93–1.47	0.18
Rotablation	3.96	0.48–32.854	0.20	2.36	0.30–18.49	0.41
GPI IIb/IIIa during PCI	1.28	0.89–1.83	0.18	1.56	1.18–2.06	0.002
TIMI before PCI	0.97	0.88–1.08	0.60	1.05	0.96–1.15	0.29
TIMI after PCI	0.29	0.28–0.32	<0.001	0.27	0.26–0.29	<0.001
Total amount of contrast, ml	0.999	0.998–1.0009	0.66	0.998	0.997–0.999	0.002
Total radiation dose, mGy.	1.0001	1.0000–1.0002	0.06	1.0001	1.0001–1.0002	<0.001

* There was no death during procedure for the positive value of the variable—the authors did not include this variable in the regression model. ASA, acetylsalicylic acid; FFR, fractional flow reserve; GPI IIb/IIIa, IIb/IIIa, glycoprotein inhibitor; IVUS, intravascular ultrasonography; LMWH, low molecular weight heparin; PCI, percutaneous coronary intervention; TIMI, thrombolysis in myocardial infarction; UFH, unfractionated heparin.

**Table 6:** Coronary anatomy, implanted stents, and complications during the procedure affecting periprocedural mortality.

Variable	Women			Men		
	OR	95% CI	*p* Value	OR	95% CI	*p* Value
RCA	1.06	0.83–1.35	0.66	0.83	0.66–1.05	0.12
LMCA	7.81	5.79–10.54	<0.001	11.99	9.56–15.02	<0.001
LAD	2.29	1.84–2.85	<0.001	1.93	1.59–2.34	<0.001
SvG	1.42	0.29–6.72	0.66	2.17	0.96–4.91	0.06
LIMA/RIMA	*			5.89	1.34–25.88	0.02
Bifurcation	0.57	0.39–0.83	0.004	0.58	0.42–0.80	0.001
DES	0.11	0.08–0.14	<0.001	0.09	0.08–0.13	<0.001
BVS	0.09	0.01–0.64	0.016	0.11	0.03–0.43	0.003
BMS	0.49	0.35–0.69	<0.001	0.40	0.29–0.56	<0.001
Number of implanted stents	1.23	1.02–1.48	0.03	1.15	0.97–1.36	0.11
DEB	1.03	0.50–2.11	0.94	0.32	0.12–0.88	0.03
Cardiac arrest during angiogram	24.55	18.74–32.16	<0.001	33.3	26.36–42.24	<0.001
Stroke	2.02	0.15–27.07	0.6			
Dissection	0.89	0.29–2.66	0.83	0.44	0.05–3.76	0.45
Bleeding at the puncture site	*			*		
Allergic reaction	*			*		
No reflow	3.37	2.43–4.67	<0.001	5.04	3.78–6.71	<0.001
Coronary artery perforation	3.09	1.44–6.65	0.004	2.02	0.69–5.88	0.2

* There was no death during procedure for the positive value of the variable—the authors did not include this variable in the regression model. BMS, bare metal stent; BVS, bioresorbable vascular scaffold; DEB, drug-eluting balloon; DES, drug-eluting stent; LAD, critical stenosis of left anterior descending artery; LMCA, critical stenosis of left main coronary artery; LIMA/RIMA, critical stenosis of left internal mammary artery/right internal mammary artery; RCA, critical stenosis of right coronary artery; SvG, critical stenosis of saphenous vein graft.

## Data Availability

The data are available on special request.

## Supplementary Materials

The following are available online at https://www.mdpi.com/article/10.3390/jcm10235716/s1, Table S1: All significant factors affecting periprocedural mortality among men. Table S2: All significant factors affecting periprocedural mortality among women. Table S3: Factors related to clinical characteristics and prehospital management. Table S4: Pharmacological and periprocedural factors. Table S5: Coronary anatomy, implanted stents, and complications during the procedure.

## References

[1] Vaccarino V., Parsons L., Every N.R., Barron H.V., Krumholz H.M. (1999). Sex-Based Differences in Early Mortality after Myocardial Infarction. N. Engl. J. Med..

[2] Vaccarino V., Parsons L., Peterson E.D., Rogers W.J., Kiefe C.I., Canto J. (2009). Sex Differences in Mortality After Acute Myocardial Infarction: Changes from 1994 to 2006. Arch. Intern. Med..

[3] Cenko E., van der Schaar M., Yoon J., Manfrini O., Vasiljevic Z., Vavlukis M., Kedev S., Miličić D., Badimon L., Bugiardini R. (2019). Sex-Related Differences in Heart Failure After ST-Segment Elevation Myocardial Infarction. J. Am. Coll. Cardiol..

[4] Cenko E., Yoon J., Kedev S., Stankovic G., Vasiljevic Z., Krljanac G., Kalpak O., Ricci B., Milicic D., Manfrini O. (2018). Sex Differences in Outcomes After STEMI: I: Effect Modification by Treatment Strategy and Age. JAMA Intern. Med..

[5] Hao Y., Liu J., Liu J., Yang N., Smith S.C., Huo Y., Fonarow G.C., Ge J., Taubert K.A., Morgan L. (2019). Sex Differences in In-Hospital Management and Outcomes of Patients with Acute Coronary Syndrome. Circulationaha.

[6] Piackova E., Jäger B., Farhan S., Christ G., Schreiber W., Weidinger F., Stefenelli T., Delle-Karth G., Kaff A., Maurer G. (2017). Gender differences in short- and long-term mortality in the Vienna STEMI registry. Int. J. Cardiol..

[7] Ben-Yehuda O., Chen S., Redfors B., McAndrew T., Crowley A., Kosmidou I., Kandzari D.E., Puskas J.D., Morice M.-C., Taggart D.P. (2019). Impact of large periprocedural myocardial infarction on mortality after percutaneous coronary intervention and coronary artery bypass grafting for left main disease: An analysis from the EXCEL trial. Eur. Heart J..

[8] Dziewierz A., Brener S.J., Siudak Z., Plens K., Rakowski T., Zasada W., Tokarek T., Bartuś K., Dudek D. (2018). Impact of On-Site Surgical Backup on Periprocedural Outcomes of Primary Percutaneous Interventions in Patients Presenting With ST-Segment Elevation Myocardial Infarction (From the ORPKI Polish National Registry). Am. J. Cardiol..

[9] Bainbridge D., Martin J., Arango M., Cheng D., Evidence-Based Peri-operative Clinical Outcomes Research (EPiCOR) Group (2012). Perioperative and anaesthetic-related mortality in developed and developing countries: A systematic review and meta-analysis. Lancet.

[10] Dudek D., Siudak Z., Grygier M., Araszkiewicz A., Dąbrowski M., Kusa J., Hawranek M., Huczek Z., Kralisz P., Roleder T. (2020). Interventional cardiology in Poland in 2019. Summary report of the Association of Cardiovascular Interventions of the Polish Cardiac Society (AISN PTK) and Jagiellonian University Medical College. Adv. Interv. Cardiol..

[11] Tavakol M., Ashraf S., Brener S.J. (2011). Risks and Complications of Coronary Angiography: A Comprehensive Review. Glob. J. Health Sci..

[12] Stehli J., Martin C., Brennan A., Dinh D.T., Lefkovits J., Zaman S. (2019). Sex Differences Persist in Time to Presentation, Revascularization, and Mortality in Myocardial Infarction Treated with Percutaneous Coronary Intervention. J. Am. Heart Assoc..

[13] Lawesson S.S., Isaksson R.-M., Thylén I., Ericsson M., Ängerud K., Swahn E., SymTime Study Group (2018). Gender differences in symptom presentation of ST-elevation myocardial infarction—An observational multicenter survey study. Int. J. Cardiol..

[14] Shah T., Haimi I., Yang Y., Gaston S., Taoutel R., Mehta S., Lee H.J., Zambahari R., Baumbach A., Henry T.D. (2021). Meta-Analysis of Gender Disparities in In-hospital Care and Outcomes in Patients with ST-Segment Elevation Myocardial Infarction. Am. J. Cardiol..

[15] Eindhoven D.C., Hilt A.D., Zwaan T., Schalij M.J., Borleffs C.J.W. (2018). Age and gender differences in medical adherence after myocardial infarction: Women do not receive optimal treatment—The Netherlands claims database. Eur. J. Prev. Cardiol..

[16] Januszek R., Siudak Z., Malinowski K.P., Wojdyła R., Mika P., Wańha W., Kameczura T., Surdacki A., Wojakowski W., Legutko J. (2020). Aspiration Thrombectomy in Patients with Acute Myocardial Infarction—5-Year Analysis Based on a Large National Registry (ORPKI). J. Clin. Med..

[17] Tokarek T., Siudak Z., Dziewierz A., Rakowski T., Krycińska R., Siwiec A., Dudek D. (2018). Clinical outcomes in nonagenarians undergoing a percutaneous coronary intervention: Data from the ORPKI Polish National Registry 2014–2016. Coron. Artery Dis..

[18] MedCalc Statistical Software (2021). Version 19.7. MedCalc Software. https://www.medcalc.org.

[19] Hannan E.L., Wu Y., Tamis-Holland J., Jacobs A.K., Berger P.B., Ling F.S.K., Walford G., Venditti F.J., King S.B. (2020). Sex differences in the treatment and outcomes of patients hospitalized with ST-elevation myocardial infarction. Catheter. Cardiovasc. Interv..

[20] Alabas O.A., Gale C.P., Hall M., Rutherford M.J., Szummer K., Lawesson S.S., Alfredsson J., Lindahl B., Jernberg T. (2017). Sex Differences in Treatments, Relative Survival, and Excess Mortality Following Acute Myocardial Infarction: National Cohort Study Using the SWEDEHEART Registry. J. Am. Heart Assoc..

[21] Rodríguez-Padial L., Fernández-Pérez C., Bernal J.L., Anguita M., Sambola A., Fernández-Ortiz A., Elola F.J. (2020). Differences in in-hospital mortality after STEMI versus NSTEMI by sex. Eleven-year trend in the Spanish National Health Service. Rev. Española Cardiol..

[22] Heer T., Hochadel M., Schmidt K., Mehilli J., Zahn R., Kuck K., Hamm C., Böhm M., Ertl G., Hoffmeister H.M. (2017). Sex Differences in Percutaneous Coronary Intervention—Insights from the Coronary Angiography and PCI Registry of the German Society of Cardiology. J. Am. Heart Assoc..

[23] Lee C.Y., Liu K.T., Lu H.T., Ali R.M., Fong A.Y.Y., Ahmad W.A.W. (2021). Sex and gender differences in presentation, treatment and outcomes in acute coronary syndrome, a 10 year study from a multi-ethnic Asian population: The Malaysian National Cardiovascular Disease Database—Acute Coronary Syndrome (NCVD-ACS) registry. PLoS ONE.

[24] Mallidi J., Lata K. (2019). Role of Gender in Dual Antiplatelet Therapy After Acute Coronary Syndrome. Curr. Atheroscler. Rep..

[25] Patti G., De Caterina R., Abbate R., Andreotti F., Biasucci L.M., Calabrò P., Cioni G., Davì G., Di Sciascio G., Golia E. (2014). Platelet function and long-term antiplatelet therapy in women: Is there a gender-specificity? A ‘state-of-the-art’ paper. Eur. Heart J..

[26] Ali M., Lange S.A., Wittlinger T., Lehnert G., Rigopoulos A.G., Noutsias M. (2017). In-hospital mortality after acute STEMI in patients undergoing primary PCI. Herz.

[27] Sliman H., Jaffe R., Rubinshtein R., Karkabi B., Zissman K., Flugelman M.Y., Zafrir B. (2019). Clinical features and outcomes of revascularization in very old patients with left main coronary artery disease. Coron. Artery Dis..

[28] Fordyce C.B., Al-Khalidi H.R., Jollis J.G., Roettig M.L., Gu J., Bagai A., Berger P.B., Corbett C.C., Dauerman H.L., Fox K. (2017). Association of Rapid Care Process Implementation on Reperfusion Times Across Multiple ST-Segment–Elevation Myocardial Infarction Networks. Circ. Cardiovasc. Interv..

[29] Nallamothu B.K., Normand S.-L.T., Wang Y., Hofer T.P., Brush E., Messenger J.C., Bradley E.H., Rumsfeld J.S., Krumholz H.M. (2015). Relation between door-to-balloon times and mortality after primary percutaneous coronary intervention over time: A retrospective study. Lancet.

[30] Squire B.T., Tamayo-Sarver J.H., Rashi P., Koenig W., Niemann J.T. (2013). Effect of Prehospital Cardiac Catheterization Lab Activation on Door-to-Balloon Time, Mortality, and False-Positive Activation. Prehospital Emerg. Care.

[31] Mehta S.R., Wood D.A., Cairns J.A. (2020). Complete Revascularization with Multivessel PCI for Myocardial Infarction. N. Engl. J. Med..

[32] Collet J.-P., Kerneis M., Lattuca B., Yan Y., Cayla G., Silvain J., Lapostolle F., Ecollan P., Diallo A., Vicaut E. (2018). Impact of age on the effect of pre-hospital P2Y12 receptor inhibition in primary percutaneous coronary intervention for ST-segment elevation myocardial infarction: The ATLANTIC-Elderly analysis. EuroIntervention.

[33] Park H.-W., Yoon C.-H., Kang S.-H., Choi D.-J., Kim H.-S., Cho M.C., Kim Y.J., Chae S.C., Yoon J.H., Gwon H.-C. (2013). Early- and late-term clinical outcome and their predictors in patients with ST-segment elevation myocardial infarction and non-ST-segment elevation myocardial infarction. Int. J. Cardiol..

[34] Rakowski T., Siudak Z., Dziewierz A., Plens K., Kleczyński P., Dudek D. (2017). Contemporary use of P2Y12 inhibitors in patients with ST-segment elevation myocardial infarction referred to primary percutaneous coronary interventions in Poland: Data from ORPKI national registry. J. Thromb. Thrombolysis.

[35] Redfors B., Furer A., Selker H.P., Thiele H., Patel M.R., Chen S., Udelson J.E., Ohman E.M., Eitel I., Granger C.B. (2020). Effect of Smoking on Outcomes of Primary PCI in Patients With STEMI. J. Am. Coll. Cardiol..

[36] Gennaro G., Brener S.J., Redfors B., Kirtane A.J., Généreux P., Maehara A., Neunteufl T., Metzger D.C., Mehran R., Gibson C.M. (2016). Effect of Smoking on Infarct Size and Major Adverse Cardiac Events in Patients with Large Anterior ST-Elevation Myocardial Infarction (from the INFUSE-AMI Trial). Am. J. Cardiol..

[37] Michas G., Karvelas G., Trikas A. (2019). Cardiovascular disease in Greece; the latest evidence on risk factors. Hell. J. Cardiol..

[38] Gurbel P.A., Bliden K.P., Logan D.K., Kereiakes D.J., Lasseter K.C., White A., Angiolillo D.J., Nolin T.D., Maa J.-F., Bailey W.L. (2013). The Influence of Smoking Status on the Pharmacokinetics and Pharmacodynamics of Clopidogrel and Prasugrel. J. Am. Coll. Cardiol..

[39] Katayama T., Iwasaki Y., Sakoda N., Yoshioka M. (2008). The Etiology of ‘Smoker’s Paradox’ in Acute Myocardial Infarction with Special Emphasis on the Association with Inflammation. Int. Heart J..

[40] Crimi G., Somaschini A., Cattaneo M., Angiolillo D.J., Piscione F., Palmerini T., De Servi S. (2017). Cigarette smoking reduces platelet reactivity independently of clopidogrel treatment in patients with non-ST elevation acute coronary syndromes. Platelets.

[41] De Luca G., Parodi G., Sciagrà R., Bellandi B., Comito V., Vergara R., Migliorini A., Valenti R., Antoniucci D. (2014). Smoking and infarct size among STEMI patients undergoing primary angioplasty. Atherosclerosis.

[42] Ferreiro J.L., Bhatt D.L., Ueno M., Bauer D., Angiolillo D.J. (2014). Impact of Smoking on Long-Term Outcomes in Patients With Atherosclerotic Vascular Disease Treated With Aspirin or Clopidogrel: Insights from the CAPRIE trial (Clopidogrel Versus Aspirin in Patients at Risk of Ischemic Events. J. Am. Coll. Cardiol..

[43] Lundergan C.F., Reiner J.S., McCarthy W.F., Coyne K.S., Califf R.M., Ross A.M. (1998). Clinical predictors of early infarct-related artery patency following thrombolytic therapy: Importance of body weight, smoking history, infarct-related artery and choice of thrombolytic regimen: The GUSTO-I experience. Global Utilization of Streptokinase and t-PA for Oc-cluded Coronary Arteries. J. Am. Coll. Cardiol..

[44] Zahger D., Cercek B., Cannon C.P., Jordan M., Davis V., Braunwald E., Shah P.K. (1995). How do smokers differ from nonsmokers in their response to thrombolysis? (The TIMI-4 trial). Am. J. Cardiol..

[45] Calvo E., Teruel L., Rosenfeld L., Guerrero C., Romero M., Romaguera R., Izquierdo S., Asensio S., Andreu-Periz L., Gómez-Hospital J.A. (2019). Frailty in elderly patients undergoing primary percutaneous coronary intervention. Eur. J. Cardiovasc. Nurs..

[46] Patel A., Goodman S.G., Yan A.T., Alexander K.P., Wong C.L., Cheema A.N., Udell J.A., Kaul P., D’Souza M., Hyun K. (2018). Frailty and Outcomes After Myocardial Infarction: Insights from the CONCORDANCE Registry. J. Am. Heart Assoc..

[47] Yoshioka N., Takagi K., Morita Y., Yoshida R., Nagai H., Kanzaki Y., Furui K., Yamauchi R., Komeyama S., Sugiyama H. (2019). Impact of the clinical frailty scale on mid-term mortality in patients with ST-elevated myocardial infarction. IJC Heart Vasc..

[48] Siudak Z., Wysocka-Dubielecka K., Malinowski K., Dziewierz A., Tokarek T., Plens K., Dudek D. (2020). Psoriasis is an independent predictor of increased risk of allergic reaction during percutaneous coronary interventions. Big data analysis from the Polish National PCI Registry (ORPKI). Cardiol. J..

