# Supplementary material for: The Effect of Periprocedural Clinical Factors Related to the Course of STEMI in Men and Women Based on the National Registry of Invasive Cardiology Procedures (ORPKI) between 2014 and 2019
Source: J Clin Med. 2021 Dec 6;10(23):5716. doi: 10.3390/jcm10235716 (PMC8658305; doi:10.3390/jcm10235716)
Supplement: Supplementary file 1 [file jcm-10-05716-s001.zip › jcm-1497923-supplementary.pdf]

Table S1. All significant factors affecting periprocedural mortality among men.

Variable	Odds ratio	95% CI	*p*
Age >65	1.57	1.32–1.87	<0.001
Diabetes	1.47	1.19–1.81	<0.001
Previous stroke	1.38	0.97–1.97	0.074
Previous MI	1.22	0.98–1.51	0.074
Smoking status	0.97	0.79–1.19	0.787
Psoriasis	2.43	1.09–5.40	0.029
Hypertension	0.74	0.62–0.89	<0.001
Kidney disease	1.29	0.91–1.81	0.148
Time from pain to first contact	1.16	1.00–1.34	0.049
OHCA	3.18	2.60–3.89	<0.001
STEMI P2Y12	0.59	0.49–0.71	<0.001
GPI IIb IIIa during angiogram	1.26	1.03–1.52	0.022
Vascular access	2.92	2.44–3.49	<0.001
GPI IIb IIIa during PCI	1.41	1.04–1.92	0.027
TIMI after PCI	0.28	0.25–0.30	<0.001
Total amount of contrast used during procedure (ml)	0.999	0.996–0.999	<0.001
Total radiation dose during procedure (mGy)	1.0001	1.0000–1.0002	0.03
LMCA	8.29	6.57–10.48	<0.001
LAD	2.18	1.84–2.58	<0.001
LIMA RIMA	3.08	0.62–15.36	0.171
Bifurcation	0.77	0.56–1.07	0.123
DES	1.05	0.82–1.33	0.713
BVS	1.74	0.42–7.24	0.449
BMS	2.13	1.55–2.93	<0.001
Cardiac arrest during angiogram	18.72	14.07–24.91	<0.001
No reflow during PCI	1.33	0.95–1.86	0.1

BMS, bare metal stent; BVS, bioresorbable vascular scaffold; DES, drug eluting stent; MI, myocardial infarction; GPI IIb/IIIa, IIb/IIIa glycoprotein inhibitor; LAD, critical stenosis of left anterior descending; LIMA/RIMA, critical stenosis of left internal mammary artery/ right internal mammary artery; LMCA, critical stenosis of left main coronary artery; OHCA, out of hospital cardiac arrest; PCI, percutaneous coronary intervention; TIMI, thrombolysis in myocardial infarction.

Table S2. All significant factors affecting periprocedural mortality among women.

Variable	Odds ratio	95% CI	*p* value
Age >65 years	1.43	1.13–1.80	0.003
Diabetes	1.43	1.17–1.76	0.001
Previous MI	1.32	1.03–1.68	0.026
Smoking	0.67	0.49–0.91	0.011
Hypertension	0.69	0.58–0.845	<0.001

Kidney disease	1.39	1.03–1.89	0.032
Time from pain to first contact	1.19	1.03–1.38	0.02
OHCA	3.60	2.86–4.54	<0.001
P2Y12	0.55	0.45–0.67	<0.001
Femoral access	2.76	2.28–3.35	<0.001
TIMI after PCI	0.31	0.28–0.34	<0.001
LMCA	4.48	3.24–6.19	<0.001
LAD	1.88	1.57–2.26	<0.001
Bifurcation	0.73	0.49–1.09	0.122
DES	0.70	0.50–0.98	0.036
BVS	0.92	0.12–7.19	0.936
BMS	1.66	1.14–2.42	0.008
Number of implanted stents	1.33	1.10–1.60	0.003
Cardiac arrest during angiogram	12.42	9.11–16.92	<0.001
No reflow	0.95	0.66–1.35	0.762
Coronary artery perforation	1.97	0.90–4.32	0.092

BMS, bare metal stent; BVS, bioresorbable vascular scaffold; DES, drug eluting stent; MI, myocardial infarction; LAD, critical stenosis of left anterior descending; LMCA, critical stenosis of left main coronary artery; OHCA, out of hospital cardiac arrest; PCI, percutaneous coronary intervention; TIMI, thrombolysis in myocardial infarction.

Table S3. Factors related to clinical characteristics and prehospital management.

**Variable**	**Total** *n*=**1465**	**Women** *n*=**663 (45,3)**	**Men** *n*=**802 (54,7)**	***p* value**
Clinical factors				
Age, median (Q1-Q3)	73 (64–82)	79 (68–85)	68 (61–78)	<0,0001
Age (>65 years)	1012 (69,1)	537 (81,0)	475 (59,2)	<0.0001
Diabetes (*n*, %)	418 (28,5)	228 (34,4)	190 (23,7)	<0.0001
Previous stroke (*n*, %)	105 (7,2)	51 (7,7)	54 (6,7)	0.4788
Previous MI (*n*, %)	300 (20,5)	124 (18,7)	176 (21,9)	0.1260
Previous PCI (*n*, %)	206 (14,1)	78 (11,8)	128 (16,0)	0.0215
Previous CABG (*n*, %)	33 (2,3)	11 (1,7)	22 (2,7)	0.1641
Smoking (*n*, %)	246 (16,8)	61 (9,2)	185 (23,1)	<0.0001
Psoriasis (*n*, %)	14 (1,0)	4 (0,6)	10 (1,2)	0.2077
Hypertension (*n*, %)	777 (53,0)	377 (56,9)	400 (49,9)	0.0077
Kidney disease (*n*, %)	132 (9,0)	73 (11,0)	59 (7,4)	0.0151
COPD (*n*, %)	38 (2,6)	14 (2,1)	24 (3,0)	0.2912
Prehospital management				
Time from pain to first contact (*n*, %)				
<12 hours	1116 (76,2)	488 (73,6)	628 (78,3)	
12–48 hours	245 (16,7)	124 (18,7)	121 (15,1)	0.1059
>= 48 hours	104 (7,1)	51 (7,7)	53 (6,6)	
Time from pain to inflation or angiogram (*n*, %)				
<12 hours	1387 (94,7)	623 (94,0)	764 (95,3)	
12–48 hours	55 (3,8)	27 (4,1)	28 (3,5)	0.4563
>= 48 hours	23 (1,6)	13 (2,0)	10 (1,2)	

Time from first contact to inflation or<br>angiogram (*n*, %)<br><12 hours<br>12–48 hours<br>>= 48 hours	 1222 (83,4)<br>170 (11,6)<br>73 (5,0)	 541 (81,6)<br>88 (13,3)<br>34 (5,1)	 681 (84,9)<br>82 (10,2)<br>39 (4,9)	0.1794
Direct transfer to cath lab (*n*, %)	437 (29,8)	201 (30,3)	236 (29,4)	0,7109
OHCA (*n*, %) STEMI	434 (29,6)	169 (25,5)	265 (33,0)	0.0016

CABG, coronary aortic bypass grafting; COPD, chronic obstructive pulmonary disease; MI, myocardial infarction; OHCA, out of hospital cardiac arrest; PCI, percutaneous coronary intervention.

Table S4. Pharmacological and periprocedural factors.

**Variable**	**Total**<br>***n*=1465**	**Women**<br>***n*=663 (45,3)**	**Men**<br>***n*=802 (54,7)**	***p* value**
Pharmacological factors				
ASA (*n*, %)	383 (26,1)	195 (29,4)	188 (23,4)	0.0097
UFH (*n*, %)	1149 (78,4)	531 (80,1)	618 (77,1)	0.1602
LMWH (*n*, %)	-	-	-	-
P2Y12 (*n*, %)	961 (65,6)	439 (66,2)	522 (65,1)	0.6514
Thrombolysis (*n*, %)	4 (0,3)	2 (0,3)	2 (0,2)	0.8487
GPI IIb/IIIa during angiogram (*n*, %)	375 (25,6)	153 (23,1)	222 (27,7)	0.0445
Bivalirudin (*n*, %)	1 (0,07)	0	1 (0,1)	0.3632
Periprocedural factors				
IVUS (*n*, %)	1 (0,07)	0	1 (0,1)	0.3632
OCT (*n*, %)	1 (0,07)	0	1 (0,1)	0.3632
Vascular access (*n*, %)<br>radial<br>femoral	476 (32,5)<br>989 (67,5)	196 (29,6)<br>467 (70,4)	280 (34,9)<br>522 (65,1)	0.0296
FFR (*n*, %)	1 (0,07)	0	1 (0,1)	0.3632
Aspiration thrombectomy (*n*, %)	200 (13,7)	85 (12,8)	115 (14,3)	0.3996
Rotablation (*n*, %)<br>GPI IIb/IIIa during PCI (*n*, %)	 113 (7,7)	 41 (6,2)	 72 (9,0)	 0.0461
TIMI before PCI (*n*, %)<br>0<br>1<br>2<br>3	1084 (74,0)<br>188 (12,8)<br>126 (8,6)<br>67 (4,6)	495 (74,7)<br>86 (13,0)<br>50 (7,5)<br>32 (4,8)	589 (73,4)<br>102 (12,7)<br>76 (9,5)<br>35 (4,4)	0.6061
TIMI after PCI (*n*, %)<br>0<br>1<br>2<br>3	530 (36,4)<br>215 (14,8)<br>255 (17,5)<br>457 (31,4)	245 (37,2)<br>94 (14,3)<br>119 (18,1)<br>201 (30,5)	285 (35,7)<br>121 (15,2)<br>136 (17,0)<br>256 (32,1)	0.8231
Total amount of contrast, ml, median (Q1-Q3)	160 (100–210)	160 (100–200)	156 (100–220)	0.4689
Total radiation dose, mGy, median (Q1-Q3)	900 (440–1644)	778 (407–1419)	1028 (471–1749)	<0.0001

ASA, acetylosalicylic acid; FFR, fractional flow reserve; GPI IIb/IIIa, IIb/IIIa glycoprotein inhibitor; IVUS, intravascular ultrasonography; LMWH, l*ow molecular weight heparin;* PCI, percutaneous coronary intervention; TIMI, thrombolysis in myocardial infarction; UFH, unfractionated heparin.

Table S5. Coronary anatomy, implanted stents, and complications during the procedure.

**Variable**	**Total** *n*=1465	**Women** *n*=663 (45,3)	**Men** *n*=802 (54,7)	*p* **value**
Coronary anatomy & implanted stents				
RCA (*n*, %)	366 (25,0)	182 (27,5)	184 (22,9)	0,0473
LMCA (*n*, %)	268 (18,3)	86 (13,0)	182 (22,7)	<0.0001
LAD (*n*, %)	835 (57,0)	386 (58,2)	449 (56,0)	0.3899
SvG (*n*, %)	10 (0,7)	2 (0,3)	8 (1,0)	0.1075
LIMA/RIMA (*n*, %)	2 (0,1)	0	2 (0,2)	0.1983
Bifurcation (*n*, %)	93 (6,3)	36 (5,4)	57 (7,1)	0.1901
DES (*n*, %)	765 (52,2)	331 (49,9)	434 (54,1)	0.1101
BVS (*n*, %)	3 (0,2)	1 (0,2)	2 (0,2)	0.6780
BMS (*n*, %)	197 (13,4)	102 (15,4)	95 (11,8)	0.0482
Number of implanted stents (*n*, %)				0.9341
0	521 (35,6)	238 (35,9)	283 (35,3)	
1	643 (43,9)	293 (44,2)	350 (43,6)	
2	231 (15,8)	100 (15,1)	131 (16,3)	
3	70 (8,4)	32 (4,8)	38 (4,7)	
DEB (*n*, %)	13 (0,9)	9 (1,4)	4 (0,5)	0.0816
Complications during the procedure				
Cardiac arrest (*n*, %)	271 (18,5)	116 (17,5)	155 (19,3)	0.3693
Stroke (*n*, %)	1 (0,07)	1 (0,2)	0	0.2714
Dissection (*n*, %)	7 (0,5)	6 (0,9)	1 (0,1)	0.0312
Bleeding at the puncture site (*n*, %)	-	-	-	-
Allergic reaction (*n*, %)	-	-	-	-
No reflow (*n*, %)	125 (8,5)	53 (8,0)	72 (9,0)	0.5025
Coronary artery perforation (*n*, %)	14 (1,0)	10 (1,5)	4 (0,5)	0.0481

BMS, bare metal stent; BVS, bioresorbable vascular scaffold; DEB, drug eluting ballon; DES, drug eluting stent; LAD, critical stenosis of left anterior descending; LMCA, critical stenosis of left main coronary artery; LIMA/RIMA, critical stenosis of left internal mammary artery/ right internal mammary artery; RCA, critical stenosis of right coronary artery; SvG, critical stenosis of saphenous vein graft.
